# A whisper from dementia

**DOI:** 10.1017/S1478951526102430

**Published:** 2026-04-20

**Authors:** Miguel Julião

**Affiliations:** Equipa Comunitária de Suporte em Cuidados Paliativos PalCo, ULS Amadora/Sintra, Amadora, Portugal

‘No human body I can touch, hold, or hug.
Spring is lost, and leaves have fallen.
My tree of life is bare and gray.
I’m old – worth a century of stories.
To mankind, I’m a “bag” – pieces of flesh tied together,
warm blood still flowing.
Lying in my bed, I ask for touch and embrace.
You give me a plastic doll, *“Here… it’s your baby.”*
You treat me as such, and I’m a century old.’

